# Magnesium supply alleviates iron toxicity-induced leaf bronzing in rice through exclusion and tissue-tolerance mechanisms

**DOI:** 10.3389/fpls.2023.1213456

**Published:** 2023-07-21

**Authors:** Toavintsoa Rajonandraina, Yoshiaki Ueda, Matthias Wissuwa, Guy J. D. Kirk, Tovohery Rakotoson, Hanna Manwaring, Andry Andriamananjara, Tantely Razafimbelo

**Affiliations:** ^1^ Laboratoire des RadioIsotopes (LRI), Université d’Antananarivo, Antananarivo, Madagascar; ^2^ Crop, Livestock and Environment Division, Japan International Research Center for Agricultural Sciences (JIRCAS), Tsukuba, Japan; ^3^ PhenoRob Cluster & Institute of Crop Science and Resource Conservation (INRES), University of Bonn, Bonn, Germany; ^4^ School of Water, Energy and Environment, Cranfield University, Cranfield, United Kingdom

**Keywords:** rice, iron toxicity, magnesium, leaf bronzing, fertilizer, RNA-Seq

## Abstract

**Introduction:**

Iron (Fe) toxicity is a widespread nutritional disorder in lowland rice causing growth retardation and leaf symptoms referred to as leaf bronzing. It is partly caused by an imbalance of nutrients other than Fe and supply of these is known to mitigate the toxicity. But the physiological and molecular mechanisms involved are unknown.

**Methods:**

We investigated the effect of magnesium (Mg) on Fe toxicity tolerance in a field study in the Central Highlands of Madagascar and in hydroponic experiments with excess Fe (300 mg Fe L^-1^). An RNA-seq analysis was conducted in a hydroponic experiment to elucidate possible mechanisms underlying Mg effects.

**Results and discussion:**

Addition of Mg consistently decreased leaf bronzing under both field and hydroponic conditions, whereas potassium (K) addition caused minor effects. Plants treated with Mg tended to have smaller shoot Fe concentrations in the field, suggesting enhanced exclusion at the whole-plant level. However, analysis of multiple genotypes showed that Fe toxicity symptoms were also mitigated without a concomitant decrease of Fe concentration, suggesting that increased Mg supply confers tolerance at the tissue level. The hydroponic experiments also suggested that Mg mitigated leaf bronzing without significantly decreasing Fe concentration or oxidative stress as assessed by the content of malondialdehyde, a biomarker for oxidative stress. An RNA-seq analysis revealed that Mg induced more changes in leaves than roots. Subsequent cis-element analysis suggested that NAC transcription factor binding sites were enriched in genes induced by Fe toxicity in leaves. Addition of Mg caused non-significant enrichment of the same binding sites, suggesting that NAC family proteins may mediate the effect of Mg. This study provides clues for mitigating Fe toxicity-induced leaf bronzing in rice.

## 1 Introduction

Rice is a staple crop for more than half the world’s population and its consumption is steadily increasing with population growth (Arouna et al., 2021). Demand is increasing particularly rapidly in Sub-Saharan Africa (SSA) where economic growth, urbanization and changes in lifestyle have increased people’s preference for rice (Wopereis et al., 2013). Despite increases in production in SSA since the 1960s (Arouna et al., 2021), yields and production are relatively low and more than 40% is imported accounting for a third of the global rice trade (FAO, 2017). Past production increase have been due to expansion of the cultivated area (Tsujimoto et al., 2019) though yields per unit area have also increased recently (Wopereis et al., 2013). Current mean yields (2.1 t ha^-1^) are far below potential yields under prevailing agro-climatic conditions (7.5–10.8 t ha^-1^) due to various abiotic and biotic constraints as well as a lack of appropriate technologies (Tsujimoto et al., 2019). Increasing productivity under adverse conditions and closing the gap between actual and potential yields are key to increasing rice productivity in SSA.

Major abiotic constraints to rice in SSA are the prevalence of highly weathered, nutrient depleted soils and the nutritional disorder iron (Fe) toxicity (Becker and Asch, 2005; van Oort, 2018). Iron toxicity occurs exclusively under submerged soil conditions where exclusion of oxygen favors generation of soluble ferrous iron (Fe(II)) from insoluble ferric iron (Fe(III)). The toxicity is exacerbated by low pH and low nutrient status, and hence it is widespread in the highly weathered soils that typify inland valleys in SSA which otherwise have great potential for rice (Becker and Asch, 2005; Kirk et al., 2022). Iron toxicity typically results in >15% rice yield loss and sometimes complete loss (Sahrawat, 2004; Audebert and Fofana, 2009). Although field management such as mid-season drainage and liming may reduce Fe(II) availability and mitigate Fe toxicity (Becker and Asch, 2005; Fageria et al., 2008), such measures are labor intensive and incur high costs. Hence, they are unlikely to be implemented in the resource-poor countries in SSA where Fe toxicity is particularly problematic. Development of tolerant germplasm is a more promising approach.

The presence of excess soluble Fe(II) in soil causes overloading of Fe in plants. Excess Fe disrupts the redox status, causes oxidative stress and foliar damage referred to as leaf bronzing, and retards growth (Aung and Masuda, 2020). Tolerance mechanisms include exclusion of excess Fe from shoots by restricting uptake into roots or by retention in roots, thus reducing root-to-shoot translocation. Genotypes lacking such Fe exclusion mechanisms may have different mechanisms to limit tissue damage despite excessive Fe concentrations (Engel et al., 2012; Matthus et al., 2015; Aung and Masuda, 2020). Such ‘includer’ mechanisms are further classified into ‘avoidance’, which involves compartmentalization of Fe in older tissues, and ‘tissue tolerance’, which involves storage of Fe in less bioactive states such as by sequestration in vacuoles and detoxification of reactive oxygen species (ROS) formed under excess Fe (Wu et al., 2017; Aung and Masuda, 2020). This suggests further genetic improvement of tolerance may be achieved by pyramiding genes for distinct tolerance mechanisms. Quantitative trait loci (QTLs) and candidate genes for Fe toxicity tolerance in rice have been identified (Dufey et al., 2009; Dufey et al., 2015a, Dufey et al., 2015b; Wu et al., 2014; Matthus et al., 2015; Diop et al., 2020; Melandri et al., 2021; Wairich et al., 2021). However, studies so far suggest that many small effect loci underlie each of the above mechanisms, as well as overall Fe toxicity tolerance.

A major problem in understanding tolerance mechanism under field conditions is interactions between Fe toxicity and the nutrient deficiencies that are prevalent in much of SSA, particularly deficiencies of phosphorus (P), potassium (K), calcium (Ca) and magnesium (Mg) (Howeler, 1973; Li et al., 2001; Gao et al., 2014; Suriyagoda et al., 2017). Of these, interaction with K uptake have been most studied. A mutant-based study (Wu et al., 2019) showed that K uptake through the K channel AKT1 restricts excess Fe uptake and mitigates excess Fe-induced leaf bronzing, and a genome-wide association study (Matthus et al., 2015) has also linked AKT1 to genotypic differences in shoot Fe concentration. Evidently K uptake reinforces Fe exclusion mechanisms. However, the interactions of Fe toxicity with other nutrients, such as Mg and Ca, have been less intensively investigated, and genes that potentially mediate the effect of these elements remain to be elucidated.

A recent meta-analysis showed that sub-optimal tissue Mg concentrations are particularly common in rice at Fe toxic sites (Kirk et al., 2022). Highly weathered soils in humid tropical regions are generally depleted in Mg due to high rates of leaching (Sun et al., 2013) and little Mg is added in fertilizers. Root-induced changes in the rhizosphere under Fe toxicity, particularly Fe oxidation and resulting acidification, will tend to decrease Mg solubility and hence its availability for plant uptake (Kirk et al., 2022). Deficiency of Mg tends to increase the levels of toxic reactive oxygen species (ROS) (Hauer-Jákli & Tränkner, 2019) typically generated under Fe toxicity (Wu et al., 2017). Links between Mg status and Fe toxicity response have also been inferred by transcriptome analysis (Kobayashi et al., 2018). This showed Mg deficiency upregulated excess Fe-inducible *OsFER2*, which encodes a Fe storage protein, and downregulated Fe sufficiency-suppressive *OsMIR* and *OsIRO2*, which encode a mitochondrial protein regulating Fe homeostasis and a key transcriptional activator regulating Fe deficiency responses, respectively (Ogo et al., 2007; Ishimaru et al., 2009; Stein et al., 2009; Aung et al., 2018). These pieces of evidence suggest that some key components of the Fe toxicity tolerance response may be inactivated at sub-optimal leaf Mg content. However, our knowledge regarding the effect of Mg supply on the Fe toxicity response and the molecular mechanisms involved remains very incomplete.

Our objectives were first, to characterize the effects of supplying Mg and K on Fe toxicity responses in a diverse set of genotypes at a strongly Fe-toxic field site in the Central Highlands of Madagascar; and second, to dissect possible mechanisms underlying the effects of Mg supply in hydroponic experiments with transcriptomic analyses.

## 2 Materials and methods

### 2.1 Field study

A field experiment was conducted with seven rice genotypes differing in their tolerance mechanism to Fe toxicity (**Table 1**; Rajonandraina et al., 2023). Plants were grown under irrigated conditions at Sambaina, Manjakandriana district in the Central Highlands of Madagascar (18°53’ S, 47°47’ E) during the wet season (December to May) in 2020-2021. The site’s soil is a Gleysol with clay loam texture, pH (H_2_O) 4.5, pH (KCl) 4.1, organic carbon 62 g kg^-1^, cation exchange capacity 2.4 cmolc kg^-1^ and total Fe 57 g kg^-1^ of which 10 g kg^-1^ is easily-soluble on soil reduction (Rakotoson et al., 2019).

**Table 1:** List of rice genotypes used in the field study.

Full Name	Short name	Origin	Response to iron toxicity
B14339E-KA-28	KA-28	Indonesia	Tolerant, inclusion
Bahia	Bahia	Spain	Tolerant, exclusion
Ciherang	Ciherang	Indonesia	Sensitive, inclusion
IR64	IR64	IRRI	Sensitive, inclusion
NERICA L-43	L-43	AfricaRice	Tolerant, exclusion
Tsipala 421	Tsipala	Madagascar	Tolerant, exclusion
X265	X265	Madagascar	Tolerant, inclusion

Origin and their putative response pattern to Fe toxicity are indicated for each genotype based on a previous study (Rajonandraina et al., 2023).

A split-plot design was implemented with eight fertilizer treatments containing all genotypes and four replications. The following treatments were applied: control (i.e. no fertilizer), Mg, NK, NKMg, NP, NPK, NPMg and NPKMg at transplanting at the following dose as in a previous study carried out in the same field (Rajonandraina et al., 2023); 50 kg N ha^-1^ as urea, 20 kg P ha^-1^ as triple superphosphate, 20 kg K ha^-1^ as potassium sulphate, and 26 kg Mg ha^-1^ as kieserite. The size of a subplot, which contained one genotype, was 1.6 m^2^ (0.8 m × 2 m). Subplots were randomized in each plot, which was further randomized in blocks of replicates. Four subplots were prepared for each genotype and treatment. Seedlings were transplanted at 21-d old with single plant per hill and a spacing of 20 cm × 20 cm between hills.

### 2.2 Sampling and measurements-field study

Sampling and measurement were done at different stages of plant growth, namely, tillering, booting, flowering and maturity. Leaf symptoms induced by Fe toxicity were visually scored as leaf bronzing score (LBS) across the whole plant canopy of each subplot as a percentage of leaf area affected on a scale from 0 (no symptoms) to 10 (100% of the leaf area affected) according to Wu et al. (2014). LBS was scored for each time point except at the maturity because of difficulties in differentiation with plant senescence. Plants were collected from two randomly selected hills per subplot, and each plant was dissected into youngest leaf (YL), middle leaves (ML), old leaves (OL) and stem/leaf sheath (ST). Flag leaves (FL) and panicles were additionally sampled at the flowering and maturing stage, respectively. The tissues were oven-dried at 60°C for 48 h until the samples were completely dry and weighed. The samples from two plants from a subplot were pooled prior to element analyses and considered as one replicate. For tissue Fe analysis, oven-dried samples were ground to a powder using a mixer mill (Retsch ZM 200, 0.2-mm sieve).

### 2.3 Hydroponic study

#### 2.3.1 Experiment 1

Seeds of Nipponbare were sterilized with 5% NaClO for 5 min and rinsed with tap water. Sterilized seeds were placed on a moistened tissue paper on a petri dish and germinated at 28°C in darkness. After 3 d, germinated seeds were placed on top of a stainless mesh tray and grown with deionized water in darkness for 2 d. Subsequently, the seedlings were exposed to natural light and kept with 0.1 mM CaCl_2_ and 12 µM FeNa-EDTA solution. After further 12 d, seedlings were transplanted to 13-L hydroponic tanks containing 0.5x concentration of Yoshida nutrient solution (Yoshida et al., 1976). After 7 d, the solution was exchanged to 1x concentration of Yoshida solution. After further 7 d, plants were transferred to hydroponic tanks containing control solution (1x Yoshida solution; Fe is provided as 36 µM FeNa-EDTA [=2 mg Fe L^-1^]) or Fe excess solution (control solution + 300 mg Fe L^-1^ as FeSO_4_.7H_2_O). A low concentration (0.1%) of melted agar (016-15817, Wako Fujifilm) was added to each container (Wang et al., 2008). The full strength (1x) Yoshida solution contained 1.42 mM NH_4_NO_3_, 0.1 mM NaH_2_PO_4_, 0.5 mM K_2_SO_4_, 1 mM CaCl_2_, 1 mM MgSO_4_, 36 µM FeNa-EDTA, 9 µM MnCl_2_, 18.5 µM H_3_BO_3_, 0.16 µM CuSO_4_, 1.5 µM ZnSO_4_ and 0.07 µM (NH_4_)_6_Mo_7_O_24_. For combinatorial treatment with different concentrations of Mg and Ca, MgSO_4_ and CaSO_4_ was added at different ratio to achieve Ca^2+^/Mg^2+^ (mM) = 1/0.2, 1/1, 1/5, and 5/1, together with normal or excess amount of Fe. After 10 d of treatment, aboveground tissues were separated into young leaves (2 most recently fully expanded leaves and emerging leaf) and the rest of tissues (old leaves + stem/leaf sheath) and used for biochemical and element analysis. LBS was measured from the 3 most recently fully expanded leaves from 5 plants in each treatment. Supplemental LED light was installed in the greenhouse due to low solar irradiation during the experiment.

#### 2.3.2 Experiment 2

Plants were grown and treated in the same manner as in Experiment 1. Plants were treated with control, excess Fe (control + 300 mg L^-1^ Fe) and excess Fe and Mg (control + 4 mM MgSO_4_ + 300 mg L^-1^ Fe) conditions. Samples for RNA extraction (n=3) were harvested after 10 d from the onset of the treatment either from the middle part of the top fully expanded leaf or whole roots. The samples were flash-frozen with liquid nitrogen and kept at -80°C until the analysis.

### 2.4 Mineral analysis

Fe concentration in dry tissue was determined as described previously (Hartmann and Asch, 2018). Briefly, Fe was extracted from pulverized dry tissues using 500 mM sodium dithionite solution, followed by colorimetric assay using 2,2’-dipyridyl. The concentration of Fe in the extract was determined by using a standard curve made with serially diluted Fe standard solution. For the measurement of other mineral elements (Ca, K, Mg), dry samples were acid-digested as reported previously (Ueda and Wissuwa, 2022; Rajonandraina et al., 2023) and measured by an inductively coupled plasma mass spectrometry (NexION 350, PerkinElmer) and inductively coupled plasma atomic emission spectrometer (ICPE-9000, Shimadzu) for field and hydroponic samples, respectively, using a standard curve made with serially diluted standard solution.

### 2.5 MDA analysis

The content of malondialdehyde (MDA) was quantified according to a previous report (Hodges et al., 1999). Briefly, 0.1% trichloroacetic acid was added to pulverized frozen samples and mixed vigorously. After centrifuging the mixture at 14,000 x g for 10 min at 4°C, 90 µL of the resultant supernatant was mixed either with solution I (0.01% 2,6-di-tert-butyl-4-methylphenol in 20% trichloroacetic acid) or solution II (solution I added with 0.65% 2-thiobarbituric acid) and heated at 95°C for 30 min. The resultant mixture was briefly centrifuged and the absorbance of the supernatant was recorded at 440, 532 and 600 nm using a microplate reader (Multiscan GO, Thermo Scientific).

### 2.6 RNA extraction and RT-PCR

Total RNA was extracted from frozen pulverized samples using RNeasy Plant Mini Kit (Qiagen) according to manufacturer’s instructions. cDNA was synthesized using the PrimeScript RT Master Mix (Perfect Real Time) kit (Takara). Quantitative RT-PCR was conducted using the CFX96 Real-Time PCR Detection System (Bio-Rad) and TB Green Premix Ex Taq II (Tli RNaseH Plus) (Takara). The expression of target genes was normalized against an internal reference gene *OsC3H38* (Höller et al., 2015).

### 2.7 RNA sequencing

Sequence library was prepared with HiSeq Standard mRNA Library Prep kit using total RNA. The resultant library was sequenced with the HiSeqX ten instrument (Illumina) and 150 bp paired-end reads were generated. Quality of the raw reads were analyzed by the FastQC software (http://www.bioinformatics.babraham.ac.uk/projects/fastqc/). Reads were trimmed by trimmomatic software (Bolger et al., 2014) using the following options; LEADING:3 TRAILING:3 SLIDINGWINDOW:4:20 MINLEN:25. Gene expression was quantified as reported previously (Pertea et al., 2016). Briefly, obtained reads were mapped to Nipponbare reference transcript (IRGSP-1.0) (Sakai et al., 2013) using HISAT2 software (Kim et al., 2015) with –min-intronlen 20 –max-intronlen 10000 options. Expression of genes was quantified using the Stringtie software (Pertea et al., 2015), yielding transcript per million (TPM) values as well as raw count data.

Differentially expressed genes were analyzed by the DESeq2 software (Love et al., 2014) using count data. PCA plot was created by plotPCA function in DESeq2 after transforming the data by the vst function.

### 2.8 *Cis*-element enrichment analysis

*Cis*-element enrichment analysis was performed as reported previously (Ashrafuzzaman et al., 2020). The promoter element (defined as upstream 1,000-bp from the start codon) for genes significantly affected by excess Fe or combination of excess Fe and Mg was obtained at Ensembl Plants Biomart website (Kinsella et al., 2011). The enrichment analysis was performed by the AME software (McLeay and Bailey, 2010), using the database of Arabidopsis *cis*-elements (O’Malley et al., 2016) as the reference. The promoter sequences of all the 18,960 genes expressed in leaves (i.e. average TPM >2) were used as control.

### 2.9 Data analysis

Statistical analyses were performed with the R program (Version 4.2.0; https://www.R-project.org/) with significance level set at *P* = 0.05. In the field experiment, treatment effects were analyzed with a mixed model ANOVA using the lmer function in the lme4 package (Bates et al., 2015). When evaluating the effect of treatment on all genotypes, treatment was considered as a fixed effect, while genotype, block (replicate), and the interaction between block and treatment factor were considered as random effects, whereas growth stage replaced genotype as random effect when evaluating the treatment effect on each genotype across growth stages. To analyze the effect of Mg, samples from NPK and NP treatments were pooled and considered as -Mg sample group, and samples from NPKMg and NPMg treatments were pooled and considered as +Mg sample group. Similarly, samples from NP and NPMg were pooled and considered as -K sample group, and samples from NPK and NPKMg were pooled and considered as +K sample group. Each of NP, NPMg, NPK, and NPKMg treatment contained 4 replicates of 7 genotypes, thus the sample size was 56 for each of -Mg, +Mg, -K and +K treatment.

In the hydroponic experiment, one-way ANOVA was used to analyze differences among different treatments. Subsequently, Tukey-Kramer *post-hoc* test was implemented to compare values of each treatment.

## 3 Results

### 3.1 Field study

#### 3.1.1 Effects of K and Mg on rice traits

We evaluated the effects of combinatorial application of N, P, K, and Mg on shoot dry weight (SDW), Fe concentration, LBS and yield. Data for all seven genotypes and four growth stages showed that P treatment had a dominant positive effect on SDW and yield, suggesting that the growth of plants was greatly limited by P availability (**Table 2**; **Figure S1**). Thus, we only considered data from the plots supplied with P in subsequent analyses.

**Table 2:** ANOVA results on the effect of genotype, P, Mg and K on Fe toxicity-related traits.

Factor	SDW	Fe concentration ^a^	LBS ^b^	Yield
Genotype	***	***	***	***
P treatment	***	ns	ns	***
Mg treatment	**	ns	***	ns
K treatment	ns	*	ns	**

a Fe concentration data derive from all examined tissues (i.e. young leaves, middle leaves, old leaves and stem/leaf sheaths).

b LBS data were only obtained from the tillering, booting and flowering stages.

The result of ANOVA is indicated as follows; ns, not significant; ns, *P* > 0.05; *, *P* < 0.05; **, *P* < 0.01: ***, *P* < 0.001.

**Figure 1:**
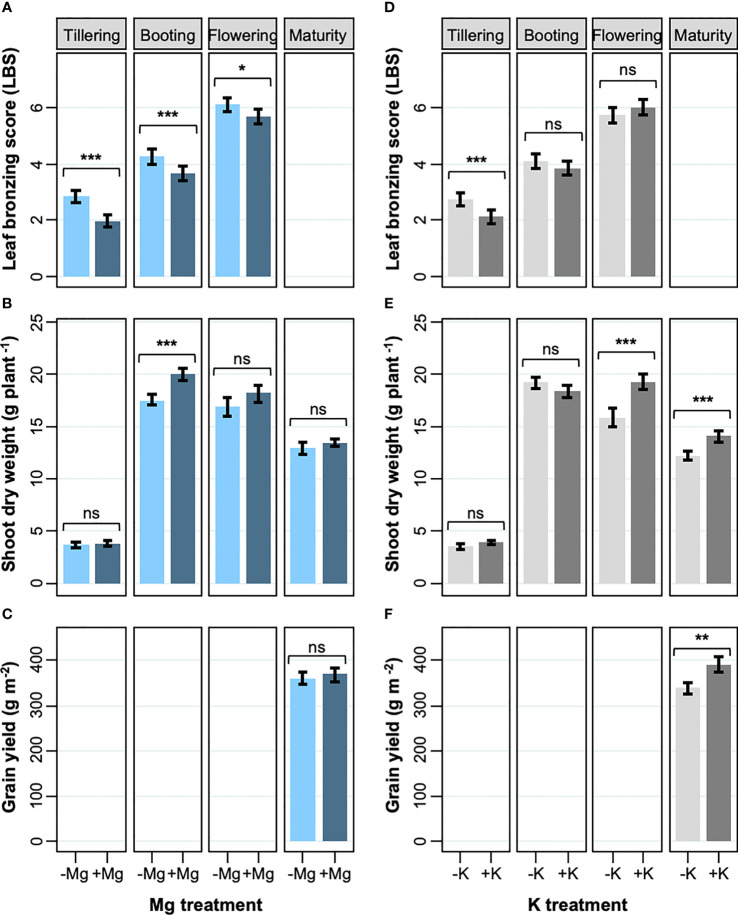
Effects of Mg and K on leaf bronzing, shoot dry weight and grain yield. **(A, D)** leaf bronzing score; **(B, E)** shoot DW; **(C, F)** grain yield for Mg treatment **(A-C)** and K treatment **(D-F)**. Data are means ± standard errors (n = 56). The result of ANOVA is indicated as follows; ns, *P* > 0.05; *, *P* < 0.05; **, *P* < 0.01; ***, *P* < 0.001.

The effects of K and Mg treatments on leaf symptoms were analyzed at different growth stages. The leaf symptoms as assessed by LBS significantly decreased with the application of Mg fertilizer by an average of 18% (**Figure 1A**). Although the decrease in LBS caused by Mg treatment lessened as the plants grew, the treatment had a consistent suppressive effect on LBS throughout the growth (34 and 6% in tillering and flowering, respectively). However, supply of Mg had no significant effect on SDW in the reproductive stages (i.e. flowering and maturity), resulting in non-significant effect on grain yield (**Figures 1B, C**; **Table 2**). The supply of K had a less striking effect on LBS at each growth stage, and the effect throughout the season was not significant (**Figure 1D**; **Table 2**). However, K addition significantly increased SDW during the reproductive stage by an average of 17% (**Figure 1E**) and this effect carried through to grain yield which was also significantly increased (+15%) (**Figure 1F**; **Table 2**). These results indicate that increased Mg supply has a beneficial effect on LBS but not on SDW or grain yield, while increased K supply had the opposite effect.

#### 3.1.2 Mg effect on shoot Fe uptake and Fe concentrations in different tissues

**Figure 2:**
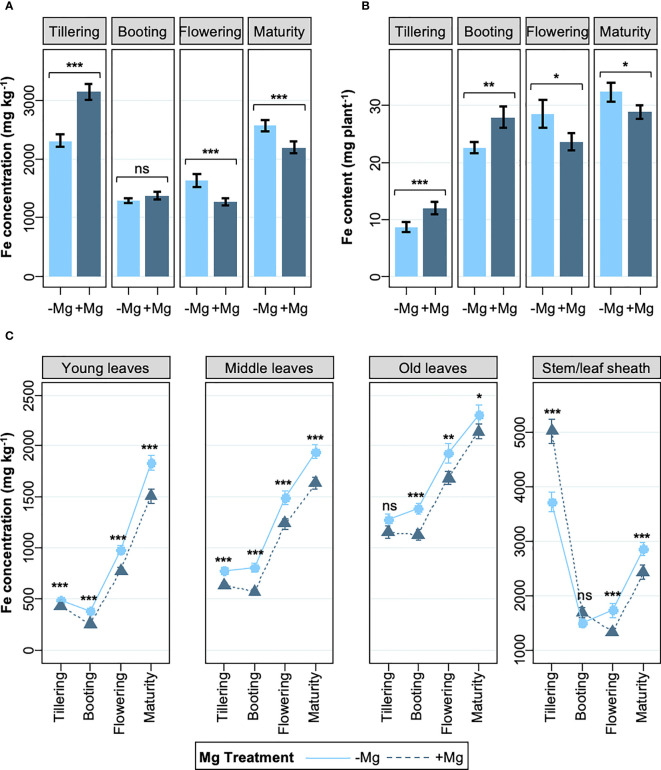
Effects of Mg on shoot Fe concentration and Fe content. **(A)** Whole shoot Fe concentration, **(B)** whole shoot Fe content, and **(C)** Fe concentrations in above-ground tissues; from tillering to maturity. Data are means ± standard errors (n = 56). The result of ANOVA is indicated as follows; ns, *P* > 0.05; *, *P* < 0.05; **, *P* < 0.01; ***, *P* < 0.001.

To get insights into the mechanism of Mg-mediated alleviation of leaf bronzing, we analyzed shoot Fe concentration and content at different growth stages. The supply of Mg significantly increased shoot Fe concentration during the vegetative stages, but the opposite effect was observed during reproductive stages (**Figure 2A**). Consistent with the effect of Mg supply on SDW and Fe concentration, aboveground Fe content (i.e. the product of SDW and Fe concentration) increased in the +Mg treatment during the vegetative and booting stages (on average by 22%), but at the reproductive growth stage the Fe content was 13% lower in the +Mg treatment (**Figure 2B**). We further assessed Fe concentrations in different plant parts. Leaf Fe concentrations increased strongly between vegetative and reproductive stages, but the +Mg treatment consistently reduced foliar Fe accumulation, except for old leaves during the vegetative stages (**Figure 2C**).

**Figure 3:**
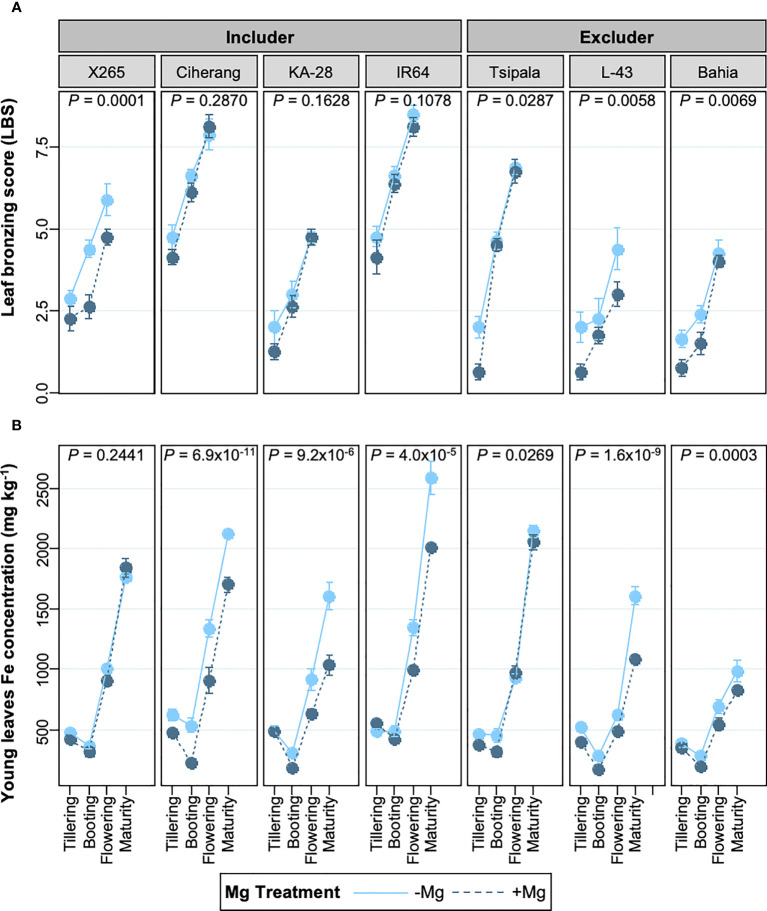
Effects of Mg on LBS and young leaves Fe concentrations in different genotypes at different growth stages. **(A)** leaf bronzing score (LBS) from tillering to flowering stage. **(B)** Fe concentration in young leaves from tillering to maturity stage. Data are means ± standard errors (n = 8). *P* values indicate the result of ANOVA analyzing the treatment effect throughout the growth stages.

We investigated if the response to Mg differed between rice genotypes depending on whether they were classified as Fe includers or excluders (Rajonandraina et al., 2023). The analysis revealed that the effect of Mg supply on LBS was genotype-specific and that the +Mg treatment alleviated leaf bronzing in four of the seven genotypes studied, but three includer-type genotypes did not benefit from Mg addition (**Figure 3A**). Curiously, these 3 includer-type genotypes had substantially reduced Fe concentrations in young leaves (26% decrease on average), whereas the reduction in Fe concentrations of young leaves was less pronounced in excluder genotypes (average decrease below 18%) (**Figure 3B**). Local rice variety X265, classified as an Fe includer, appears to be an exception, as Mg addition did not lower Fe concentrations in young leaves while significantly decreasing LBS. We analyzed if these changes were due to an increase in tissue Mg concentrations. Mg concentrations in young leaves at the booting stage were not significantly affected by Mg addition in any of the examined genotypes (**Figure S2**), raising the possibility that the effect of Mg was likely through altered rhizosphere processes or interaction with other nutrients (**Figure S2**), at least at this growth stage.

### 3.2 Hydroponic study

#### 3.2.1 Mg, but not Ca, alleviates Fe toxicity in hydroponic conditions

**Figure 4:**
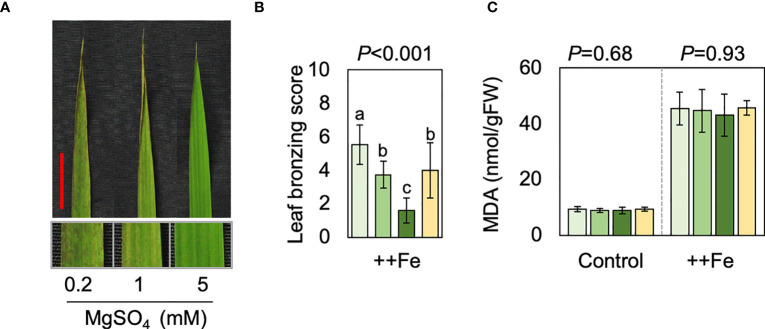
Effects of Mg and Ca in hydroponics. **(A)** Representative image of top fully expanded leaf after 10 d of excess Fe treatment in the presence of different concentrations of Mg. The scale bar indicates 2 cm. The below panels indicate magnified image. **(B, C)** LBS **(B)** and MDA content **(C)** are shown for control (2 mg L^-1^ Fe) and excess Fe (300 mg L^-1^ Fe) treatments. In B and C, one-way ANOVA was conducted for each treatment and the resultant *P* value is shown on the top of each graph. Different alphabets indicate that the values are significantly different. Data are means ± standard deviations (n=4-5).

We conducted hydroponic studies to reveal potential effects of Mg and reveal associated molecular mechanisms. Plants received normal or excess Fe (2 and 300 mg Fe L^-1^, respectively), together with different concentrations of Mg. Root morphology and iron plaque formation were not substantially affected by the different Mg concentrations (**Figure S3**). We also note that additional Mg did not substantially affect the pH or the redox potential of the solution (**Figure S4**), suggesting that Mg treatment did not affect Fe availability in the hydroponic solution. Supplying additional Mg reduced LBS in the three newest fully expanded leaves, while Mg deficiency had an opposite effect (**Figures 4A, B**). On the other hand, increasing the concentration of Ca, which like Mg is predominantly present as a divalent cation in the hydroponic solution, did not alleviate leaf bronzing (**Figure 4B**). The content of malondialdehyde (MDA), which is a biomarker for oxidative stress, was measured in new leaves, which we defined as the emerging and two newest fully expanded leaves. The MDA content increased drastically under Fe toxicity but ANOVA indicated differences between Mg or Ca treatments were not significant (**Figure 4C**).

**Figure 5:**
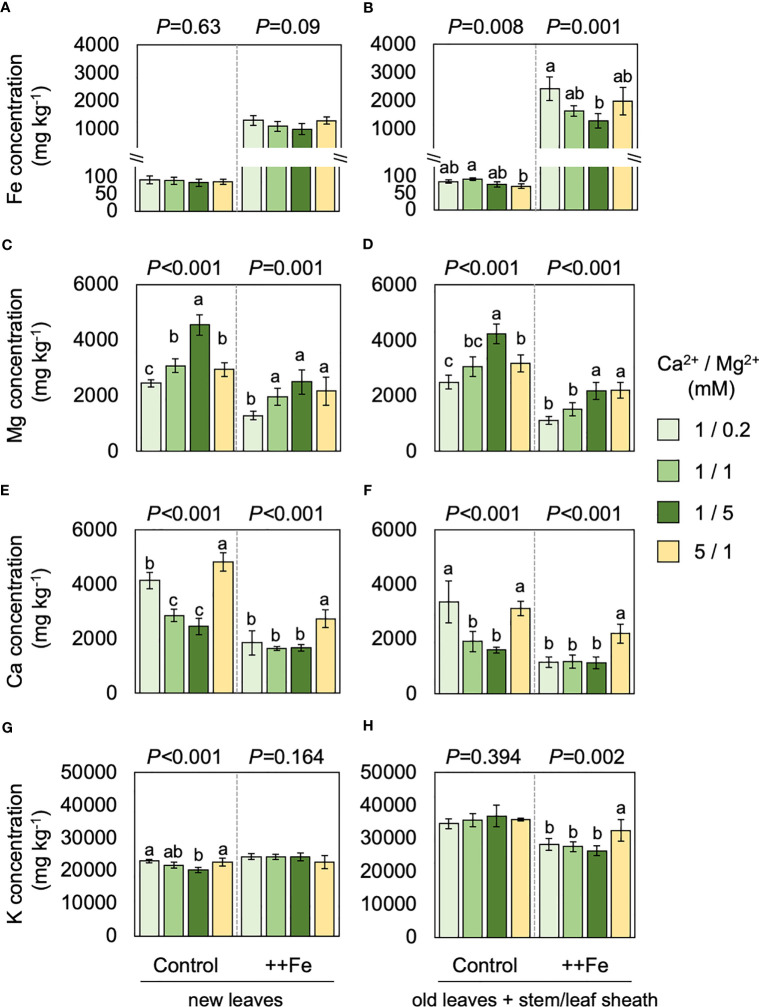
Effects of Fe, Mg and Ca on shoot element concentrations. Concentrations of Fe **(A, B)**, Mg **(C, D)**, Ca **(E, F)** and K **(G, H)** treated with different levels of Fe, Mg and Ca. Data for new leaves **(A, C, E, G)** and old leaves and stem/leaf sheath **(B, D, F, H)** are shown. One-way ANOVA was conducted for each treatment and the resultant *P* value is shown on the top of each graph. Different alphabets indicate that the values are significantly different. Data are means ± standard deviations (n=4-5).

Element concentrations in shoot tissues were substantially affected by different treatments. Fe concentrations in new leaves were not significantly affected by Ca or Mg treatments (**Figure 5A**) but in old leaves and stem/leaf sheath the addition of Mg caused a reduction in tissue Fe concentrations whereas the Ca treatment did not have a substantial effect (**Figure 5B**). Excess Fe drastically reduced tissue Mg concentrations but different levels of Mg significantly affected Mg concentrations both in new leaves and the rest of shoot (i.e. old leaves and stem/leaf sheaths), irrespective of Fe conditions (**Figures 5C, D**). Ca concentrations were also reduced by excess Fe but drastically increased by additional Ca supply. Increment in Mg supply negatively affected Ca concentrations under normal Fe condition but not under excess Fe (**Figures 5E, F**). Concentrations of K was rather stable and affected only to a small extent by different levels of Fe, Mg and Ca treatments (**Figures 5G, H**).

#### 3.2.2 Genes and *cis*-elements involved in Mg-mediated stress alleviation

**Figure 6:**
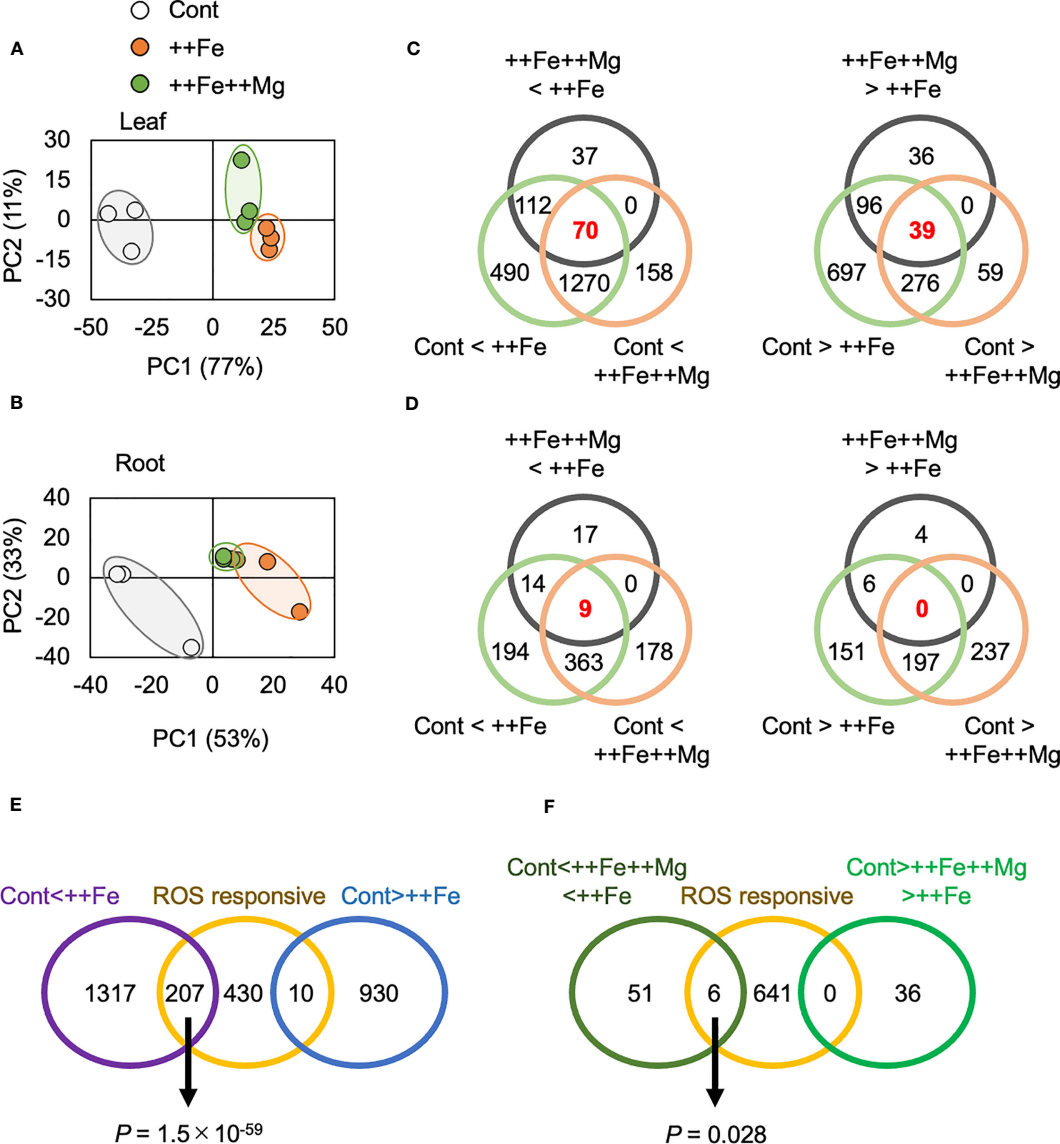
Effects of Mg on gene expression. **(A, B)** The result of PCA analysis using gene expression data in leaf **(A)** and root **(B)**. **(C, D)** Venn diagrams showing the number of differentially expressed genes in each pair of comparison in leaf **(C)** and root **(D)**. **(E, F)** Venn diagrams showing the number of differentially expressed genes and previously suggested ROS-responsive genes. Fisher’s exact test was performed, and *P* value is indicated for significantly overlapped regions.

We performed an RNA-seq analysis using root and leaf tissue of plants grown under the Cont, ++Fe and ++Fe++Mg conditions (**Table S1**). The PCA analysis clearly separated clusters for Cont, ++Fe and ++Fe++Mg for leaf samples (**Figure 6A**) but the separation between ++Fe and ++Fe++Mg sample groups was less clear for roots (**Figure 6B**). Considering that Mg alleviates Fe toxicity, we reasoned that the key genes involved in Mg-mediated effects would exhibit one of the following expression patterns; Cont < ++Fe++Mg < ++Fe or Cont > ++Fe++Mg > ++Fe. Thus, genes that fulfill these criteria in leaf or root samples were identified. Among 18,960 genes expressed in leaves (i.e. average TPM > 2), 219 and 171 genes were down- and up-regulated in ++Fe++Mg compared with ++Fe, respectively (**Figure 6C**). Among them, 70 and 39 genes exhibited the expression pattern of Cont < ++Fe++Mg < ++Fe and Cont > ++Fe++Mg > ++Fe, respectively (**Figure 6C**; **Table S2**). In roots, among 20,375 expressed genes, only a very small number of genes exhibited the pattern of Cont < ++Fe++Mg < ++Fe and Cont > ++Fe++Mg > ++Fe (9 and 0 genes, respectively) (**Figure 6D**; **Table S2**). We conclude that the function of Mg to mitigate Fe toxicity is *via* mechanisms specific to shoot rather than a root tissue.

To examine if Mg treatment mitigates Fe-induced oxidative stress, we further compared our RNA-seq results with two previously reported transcriptome studies conducted under ozone stress, which causes oxidative stress directly in leaves (Frei et al., 2010; Ashrafuzzaman et al., 2018). 15,870 genes were detected in both previous studies and the current study, and among these, 647 genes were defined as ‘ROS-responsive’ because they were similarly affected by ozone stress in both previous studies (Frei et al., 2010; Ashrafuzzaman et al., 2018). These ROS-responsive genes overlapped with 207 ++Fe-induced and 10 ++Fe-suppressed genes, and the degree of overlap between ROS-responsive and ++Fe-inducible genes was highly significant (*P* = 1.5 × 10^-59^ by Fisher’s exact test), confirming that excess Fe causes oxidative stress in leaves (**Figure 6E**). On the other hand, only 6 ROS-responsive genes (0.9%) overlapped with genes whose expression follows Cont < ++Fe++Mg < ++Fe in leaves, and the significance level of the overlap was much lower (*P* = 0.028) (**Figure 6F**).

**Figure 7:**
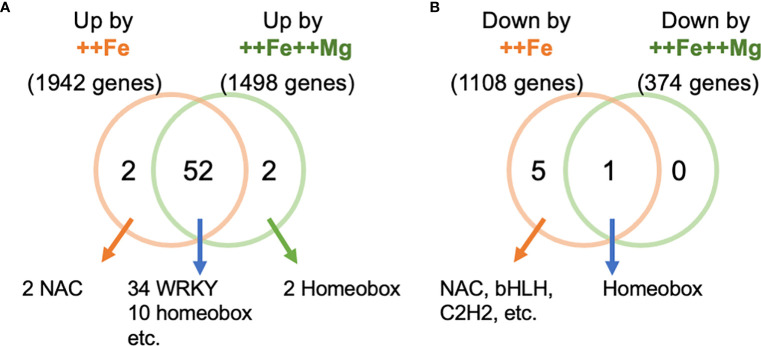
*Cis*-element enrichment analysis. The number of enriched *cis*-elements from the genes upregulated **(A)** and downregulated **(B)** genes by ++Fe and ++Fe++Mg treatments.

We next searched for potential regulatory factors that mediate the effect of Mg in leaves. We identified promoter elements that are enriched among differentially expressed genes in leaves under ++Fe or ++Fe++Mg conditions. 52 *cis-*elements were commonly enriched by both treatments (i.e. among 1,942 and 1,498 genes upregulated by ++Fe and ++Fe++Mg treatment, respectively), such as those for WRKY and homeobox transcription factors (**Figure 7A**; **Table S3**). Two homeobox binding motifs and two NAC binding sites were enriched exclusively in ++Fe++Mg and ++Fe treatments, respectively. Among 1,108 and 374 genes downregulated by ++Fe and ++Fe++Mg treatments, only one homeobox binding site was enriched in both treatments, and 5 *cis*-elements, including the binding site for NAC, bHLH and C2H2 transcription factors, were enriched only by ++Fe treatment, while no element was specifically detected by ++Fe++Mg treatment (**Figure 7B**; **Table S3**).

## 4 Discussion

### 4.1 Fe exclusion mechanisms conferred by Mg supply

Plant responses to an imbalance in the availability of a nutrient are dependent on the status of other mineral elements. For instance, the strength of P deficiency responses is affected by the availability of N and Fe (Balzergue et al., 2017; Hu et al., 2019; Ueda et al., 2020). Fe toxicity is observed at tissue Fe concentrations ranging from 300 to 2000 ppm (Dobermann and Fairhurst, 2000) and this wide range of critical tissue concentrations is likely due to interactions between excess Fe and deficiencies of other nutrients such as P, K, Ca, Zn and Mg. Such deficiencies may be exacerbated by excess Fe-induced changes in the rhizosphere coupled with unfavorable soil properties such as low cation exchange capacity and pH buffer power (Becker and Asch, 2005; Kirk et al., 2022). Increased availability of these elements consistently mitigates symptoms caused by Fe toxicity (Benckiser et al., 1984; Sahrawat, 2004). For instance, an application of dolomite, which is a mixture of Mg and Ca, effectively mitigated Fe toxicity-triggered reductions in growth and yield of rice in an acid Fe toxic soil in Sri Lanka (Suriyagoda et al., 2017). Similarly, the ratio of available Fe to other divalent cations (i.e. Ca, Mg, Mn) were more closely associated with the degree of yield reduction than Fe concentration alone in Fe toxic acid sulphate soils in Thailand (Moore et al., 1990). These studies would suggest the benefit of increased supply of divalent cations lies in lowering excess Fe uptake, possibly by direct competition, or increasing their supply offsets any negative effects of their deficiency on Fe toxicity responses. Our data suggest that in some genotypes Mg supply helps Fe exclusion mechanisms (**Figure 3**), which could be explained by multiple factors. Tadano (1975) reported that reduced Mg supply lowered the Fe exclusion capacity of roots and increased the ratio of Fe translocated to shoots in culture solution, suggesting that Mg decreases Fe uptake or excess root-to-shoot translocation or both. Additionally, increased Fe concentration in stems by Mg treatment (**Figure 2C**) may form another layer of Fe-exclusion mechanism, as suggested previously (Engel et al., 2012). These effects of Mg on Fe uptake, root-to-shoot translocation and retention in stems could be regarded as a general function of Mg under Fe toxicity, as seen by a negative correlation between leaf Fe and Mg concentrations in a pot study using Fe toxic soils (Genon et al., 1994).

### 4.2 Mg-induced tissue-tolerance mechanisms

Even though Fe exclusion mechanisms are strengthened, some pieces of evidence suggested tissue-tolerance mechanisms conferred by Mg supply. It was suggested that the concentration ratio of Fe to Mg + Ca in flag leaves, rather than the concentration of Fe *per se*, is a good indicator for leaf bronzing under Fe toxicity (Sylla, 1994). This implies that Ca and Mg may confer at least some tissue tolerance. Our observations in field and hydroponic conditions both indicated attenuated leaf bronzing formation by Mg supply without a concomitant reduction in Fe concentration (X265 in **Figures 3**–**5**). A previous study also showed that Mg deficiency did not have a substantial effect on leaf Fe concentration but did induce genetic responses similar to those observed under Fe toxicity (Kobayashi et al., 2018). This suggests that Mg supply may also support tissue-based tolerance mechanisms. The hypothesis that increased Mg supply confers tissue tolerance is also supported by genotypic differences in the benefit provided by Mg treatment. Our data show that the includer genotypes benefitted to a lesser extent from Mg fertilization than the excluder genotypes (**Figure 3A**). This implies that Mg supply may provide tissue tolerance to plants that already possess exclusion mechanisms, i.e., an additive effect. However, the associated mechanisms underlying increased tissue tolerance conferred by Mg supply might be different in field and hydroponic studies, since we did not observe significant increase in Mg concentrations in young leaves at the booting stage in the field study. It implies that altered rhizospheric chemical processes or secondary effects on the uptake of other nutrients (**Figure S2**), rather than an increase in foliar Mg concentrations, could explain this phenomenon, at least at this growth stage.

### 4.3 Possible physiological and genetic factors underlying Mg-induced tissue-tolerance mechanisms

Contrary to the field study, additional Mg supply increased tissue Mg concentrations in hydroponic study (**Figure 5**), which clearly suggests that tissue tolerance is conferred by increased tissue Mg concentrations. Deficiency of Mg disrupts Fe storage in leaf cell vacuoles (Kobayashi et al., 2018) and additional Mg supply might increase storage of Fe in vacuoles that is otherwise kept in cytosol or other compartments in a toxic state. Interestingly, in our hydroponic experiment, the reduction in leaf bronzing was not accompanied by a decrease of MDA, a biomarker for oxidative stress. These data indicate that the leaves of plants supplied with excess Mg perceive a similar extent of oxidative stress but form less leaf bronzing, suggesting that Mg specifically suppresses the process of cell death caused by oxidative stress. Thus, the underlying mechanism of Mg effects observed in our study is not *via* reduced oxidative stress as previously suggested (Tränkner et al., 2016; Hauer-Jákli and Tränkner, 2019). This is further supported by the gene expression data showing that most of the genes affected by Mg were not related to oxidative stress (**Figures 6E, F**).

The promoters of Fe-inducible genes were enriched with *cis*-elements such as WRKY, NAC and Homeobox-binding motifs as previously suggested (Kakei et al., 2021) (**Figure 7A**; **Table S3**). The binding sites for WRKY transcription factors were also enriched among the genes that were induced by the ++Fe++Mg treatment, suggesting that WRKY family proteins may not play pivotal roles in Mg-dependent alleviation of symptoms (**Figure 7A**; **Table S3**). In Arabidopsis, half of Mg deficiency-responsive genes are also responsive to ABA (Tanoi and Kobayashi, 2015), suggesting a close link between ABA and Mg status. Interestingly, some members of NAC transcription factor family that likely mediate the effect of Mg are induced by ABA and play central roles in the ABA signal transduction pathway (Todaka et al., 2012). Our results also show that many Mg-responsive genes (i.e. genes that exhibit the expression patterns of Cont <++Fe++Mg < ++Fe or Cont > ++Fe++Mg > ++Fe) are ABA, salinity or drought-related genes; Among 70 genes whose expression pattern follows Cont < ++Fe++Mg < ++Fe in leaf, *OsEBP89*, *bHLH035*, *ONAC60*, *MYBR1*, *PIP1;3*, and *RAV2* are known to be involved in drought or salinity stress response and tolerance (Fang et al., 2014; Duan et al., 2016; Yin et al., 2017; Chen et al., 2018; Liu et al., 2020; Zhang et al., 2020). These genes also contained some NAC transcription factors (i.e. *NAC15*, *ONAC60*, *NAC58*, *NAC121* and *NAC103*). Further investigations are necessary to identify the key gene(s) mediating the effect of Mg and elucidate the relationship between Mg, ABA and the causal gene under Fe toxicity conditions.

### 4.4 Beneficial effects of Mg on mineral stresses

Optimal Mg supply is known to counteract several soil-related stresses. For instance, Mg confers tolerance to aluminum (Al) stress in various plant species (Rengel et al., 2015). In rice, Mg alleviates Al stress partly by reducing Al uptake and oxidative stress (Pandey et al., 2013), although the physiological pathways affected by Mg may vary in different plant species (Silva et al., 2001a). Mg also confers tolerance of salinity stress in rice (Chen et al., 2017). In both cases, Mg uptake, rather than the contact of roots with high Mg concentration, is the key factor, as shown by the attenuated tolerance in mutant lines lacking functional MGT1 which is a plasmamembrane-localized major Mg transporter (Chen et al., 2012; Chen et al., 2017).

Contrary to the effect of Mg, additional Ca application in our hydroponic study did not significantly affect leaf bronzing or Fe accumulation. This could be partly due to the similarity of the ionic radii of Fe^2+^ (77 pm) and Mg^2+^ (72 pm) compared with Ca^2+^ (100 pm). Similarity in radii may mean that Mg^2+^ competes with Fe^2+^ better than Ca^2+^ for binding with other molecules and mitigate the deleterious effects of Fe to confer tissue tolerance. Similar Mg-specific effects were also observed under Al stress. A previous investigation in soybean suggested that Al toxicity was specifically mitigated by Mg^2+^ but not by similar concentration of Ca^2+^ (Silva et al., 2001a; Silva et al., 2001b), consistent with our observation that the positive effect of Mg^2+^ is not simply due to the ionic strengths of competing divalent cations. Interestingly, the expression of *MGT1* is induced both in roots and leaves under Fe toxicity (**Table S1**). Thus, it is possible that upregulation of *MGT1* and accompanying increase of Mg uptake might be an intrinsic mechanism that plants possess to cope with Fe toxicity, as well as other edaphic stresses such as salinity and Al toxicity. *MGT1* is found within a QTL explaining Al stress tolerance (Yamaji et al., 2009). Intriguingly, a QTL for Fe toxicity-induced leaf bronzing (*qFETOX1-2*) was previously found close to the position of *MGT1* (37.7 Mb on chromosome 1) (Wu et al., 2014), which is also close to a QTL for foliar Mg concentration (Norton et al., 2010). Whether allelic variation exists at *MGT1* locus, and if that is the cause of genotypic variation of leaf bronzing, needs further investigation.

### 4.5 Conclusions and future perspectives

We have shown in field and hydroponic experiments that increasing the supply of Mg to rice roots improves Fe toxicity tolerance *via* exclusion- and tissue tolerance-based mechanisms. Evidence from transcriptome analysis indicated the involvement of several genes expressed in shoot tissue that potentially mediate the Mg effect, and of particular relevance seem to be the altered expression levels of genes containing the binding sites for NAC transcription factors. Previous studies showed more than three-fold variation in leaf Mg concentration among diverse rice accessions grown under control conditions (Chen and Ma, 2013; Yang et al., 2018), suggesting scope for improvement of the Mg status in rice plants through a breeding approach. Whether utilizing potential allelic variation of *MGT1*, as well as increased foliar Mg concentration effectively reduces Fe toxicity in the field need to be investigated further.

## Data availability statement

The datasets presented in this study can be found in online repositories. The names of the repository/repositories and accession number(s) can be found below: NCBI SRA accession number: PRJNA936902 under the following link: https://www.ncbi.nlm.nih.gov/sra/PRJNA936902.

## Author contributions

TRaj, MW and GK conceived and designed the field experiment. TRaj, MW, GK and TRak conducted field experiment. TRaj and TRak obtained phenotypic data from the field experiment. HM conducted element analyses of field samples. MW, GJDK, TRak, AA and TRaz supervized the field experiment. TRaj, MW and TRak analyzed data from the field experiment. YU conceived and conducted the hydroponic experiments and RNA-seq and analyzed these data. TRaj and YU wrote the manuscript draft and prepared figures. All authors edited and approved the final manuscript.
