# Supplementary material for: Magnesium supply alleviates iron toxicity-induced leaf bronzing in rice through exclusion and tissue-tolerance mechanisms
Source: Front Plant Sci. 2023 Jul 21;14:1213456. doi: 10.3389/fpls.2023.1213456 (PMC10403268; doi:10.3389/fpls.2023.1213456)
Supplement: Supplementary file 1 [file DataSheet_1.pdf]

SUPPLEMENTARY FIGURES

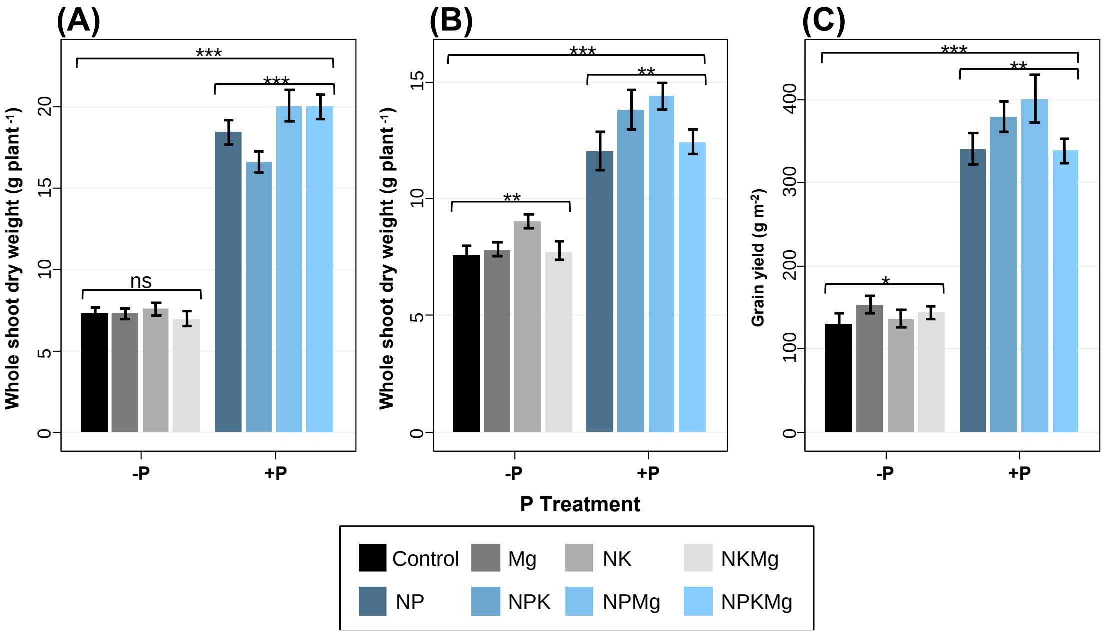

Fig. S1. Effects of combinatorial fertilizer application on growth and yield.

Shoot dry weight at booting (A) and maturity stages (B), as well as grain yield (C) are shown for different treatments. Data are means ± standard errors (n = 28). The result of ANOVA is indicated as follows; ns, $P > 0.05$; *, $P < 0.05$; **, $P < 0.01$; ***, $P < 0.001$.

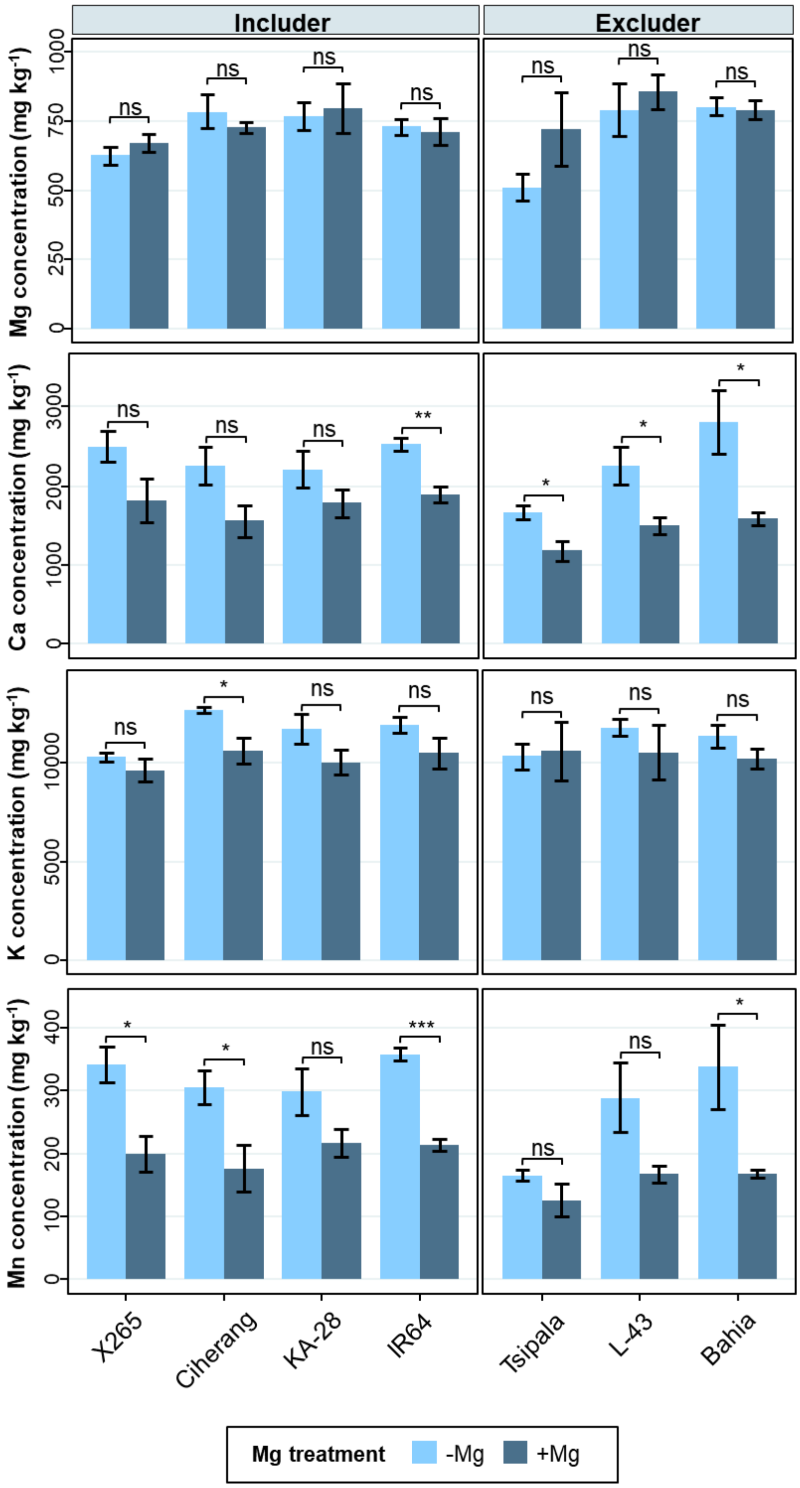

Fig. S2. Effects of Mg on element concentrations in young leaves at the booting stage in the field.

The concentrations of Mg, Ca, K and Mn in young leaves at the booting stage are shown for 7 genotypes. Data are means ± standard errors (n = 4). The significance level is shown as follows; ns, $P > 0.05$; *, $P < 0.05$; **, $P < 0.01$; ***, $P < 0.001$.

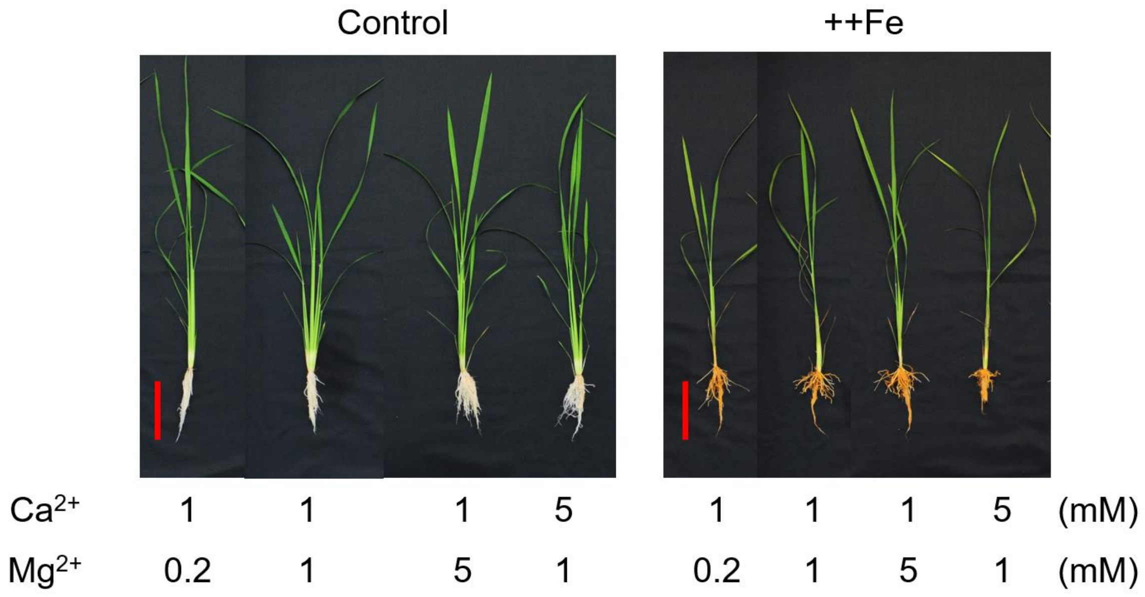

Fig. S3. Effects of Mg and Ca treatment on morphology and root plaque formation.

Representative images of plants grown with different concentrations of Fe, Ca and Mg are shown.

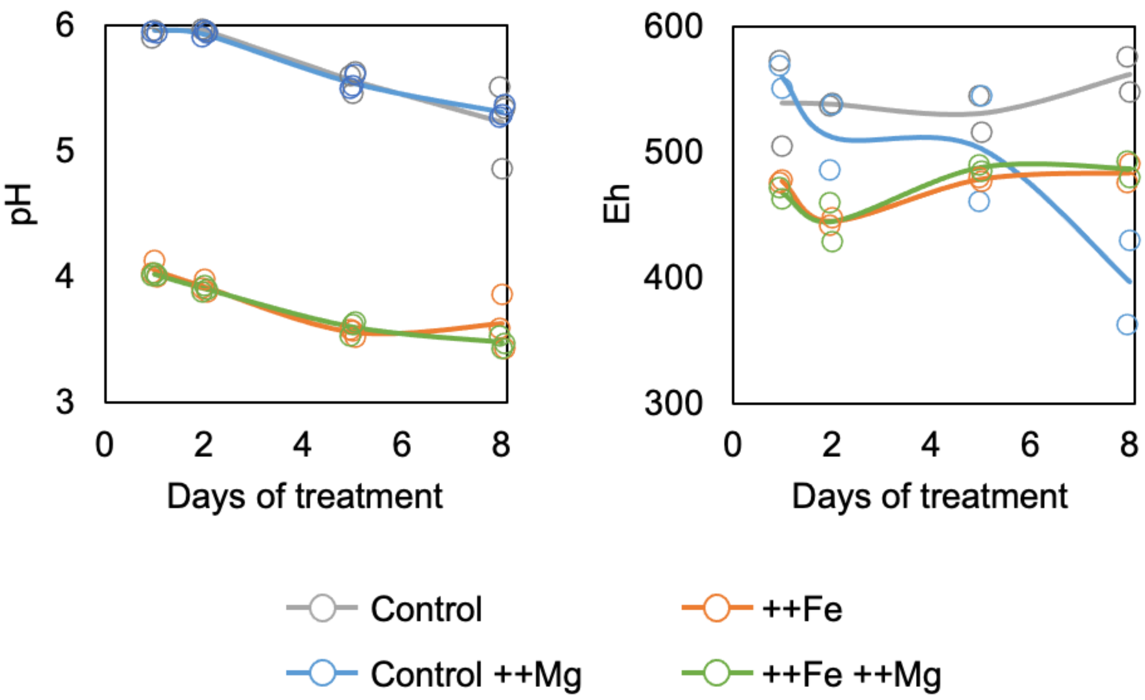

Fig. S4. Effects of treatments on pH and Eh in hydroponics.
